# TME10/380: Remote Transmission of Radiological Images by means of Intranet/Internet Technology

**DOI:** 10.2196/jmir.1.suppl1.e117

**Published:** 1999-09-19

**Authors:** F Sicurello, R Pizzi

**Affiliations:** ^1^Istituto Nazionale Neurologico C. Besta, MilanoItaly

**Keywords:** Web, Booking

## Abstract

At the Istituto Nazionale Neurologico C. Besta in Milano a network architecture has been developed to connect computers and diagnostic modalities, based on Intranet technology in order to allow the hospital to have an external access through the Internet. The Internet technology has become the "glue" that allows to link different computers and to develop applications able to work independently from the hardware/software platform. Using a PACS (Picture Archiving and Communication System) system integrated to the diagnostic modalities by means of the standardized DICOM image format, the digital radiological images can be transferred, displayed and processed on special visualization workstations all around the hospital. From the workstations the same images can be transferred in DICOM format to a teleconsulting workstation. In fact the hospital is involved in a national project for the remote connection between many Italian hospitals. This national network is linked to already developed regional networks like the Toscana MAN and the ATM Sirius Network. Some links are performed directly in ATM (155 Mbps), others are based on CDN (Direct Numerical Connection, 2Mbps), others are simply based on ISDN connections. The system allows to make it simpler and faster the already established daily exchange of radiological reports between the involved hospitals, especially from Istituto Nazionale Neurologico and Istituto Nazionale deiTumori. All the actions performed by the radiologist are translated by the software into "events" and replied to the remote workstation and vice-versa. In this way the radiologists can see each others, speak together and act in real time on a common "board" of diagnostic images, each one with his own pointer. The adopted technology is evolving on a system based on a web architecture and Java applications, useful for small clinical centers not endowed with expensive information systems. These centers will be able to get consulting performances by the excellence centers, making available accurate diagnoses and therapy protocols.

